# Large intragenic deletion of *CDC73* (exons 4–10) in a three-generation hyperparathyroidism-jaw tumor (HPT-JT) syndrome family

**DOI:** 10.1186/s12881-017-0445-0

**Published:** 2017-08-03

**Authors:** Vito Guarnieri, Raewyn M. Seaberg, Catherine Kelly, M. Jean Davidson, Simon Raphael, Andrew Y. Shuen, Filomena Baorda, Orazio Palumbo, Alfredo Scillitani, Geoffrey N. Hendy, David E. C. Cole

**Affiliations:** 1 Medical Genetics, IRCCS Casa Sollievo della Sofferenza Hospital, San Giovanni Rotondo, Italy; 2 Department of Otolaryngology - Head and Neck Surgery, University of Toronto, Toronto, ON Canada; 3 Department of Medicine, University of Toronto, Toronto, ON Canada; 4 Division of Endocrinology, Women’s College Hospital, Toronto, ON Canada; 5 Department of Otolaryngology, Head & Neck Surgery, Sunnybrook Health Sciences Centre, Toronto, ON Canada; 6 Department of Anatomic Pathology, Sunnybrook Health Sciences Centre, Toronto, ON Canada; 7 Departments of Laboratory Medicine and Pathobiology, Medicine and Genetics, University of Toronto, Toronto, ON Canada; 8 Endocrinology, IRCCS Casa Sollievo della Sofferenza Hospital, San Giovanni Rotondo, Italy; 9 Metabolic Disorders and Complications, McGill University Health Centre-Research Institute, Montreal, QC Canada; 10 Departments of Medicine, Physiology and Human Genetics, McGill University, Montreal, QC Canada

**Keywords:** Parathyroid carcinoma, HPT-JT syndrome, Germline, CDC73

## Abstract

**Background:**

Inactivating mutations of *CDC73* cause Hyperparathyroidism-Jaw Tumour syndrome (HPT-JT), Familial Isolated Hyperparathyroidism (FIHP) and sporadic parathyroid carcinoma. We conducted *CDC73* mutation analysis in an HPT-JT family and confirm carrier status of the proband’s daughter.

**Methods:**

The proband had primary hyperparathyroidism (parathyroid carcinoma) and uterine leiomyomata. Her father and daughter had hyperparathyroidism (parathyroid adenoma) but no other manifestations of HPT-JT. *CDC73* mutation analysis (sequencing of all 17 exons) and whole-genome copy number variation (CNV) analysis was done on leukocyte DNA of the three affecteds as well as the proband’s unaffected sister.

**Results:**

A novel deletion of exons 4 to 10 of *CDC73* was detected by CNV analysis in the three affecteds. A novel insertion in the 5’UTR (c.-4_-11insG) that co-segregated with the deletion was identified. By in vitro assay the 5’UTR insertion was shown to significantly impair the expression of the parafibromin protein. Screening for the mutated *CDC73* confirmed carrier status in the proband’s daughter and the biochemistry and ultrasonography led to pre-emptive surgery and resolution of the hyperparathyroidism.

**Conclusions:**

A novel gross deletion mutation in *CDC73* was identified in a three-generation HPT-JT family emphasizing the importance of including screening for large deletions in the molecular diagnostic protocol.

**Electronic supplementary material:**

The online version of this article (doi:10.1186/s12881-017-0445-0) contains supplementary material, which is available to authorized users.

## Background

Primary hyperparathyroidism (PHPT) is a common endocrine disorder affecting up to 2% of individuals over the age of 55 years [1]. It is caused by solitary benign adenoma in 80–85%, hyperplasia in 10–15%, and parathyroid carcinoma in less than 1%. In up to 10% of cases PHPT is part of a familial syndrome such as multiple endocrine neoplasia (MEN) types 1 or 2, hyperparathyroidism-jaw tumor syndrome (HPT-JT), familial isolated hyperparathyroidism (FIHP) or familial hypocalciuric hypercalcemia [2]. In the HPT-JT syndrome, carcinomas account for approximately 15% of the parathyroid tumors [3].

The most common manifestations of the autosomal dominant HPT-JT syndrome are parathyroid tumours and ossifying fibromas of the maxilla and mandible. Patients may also develop renal abnormalities and uterine tumors [4]. The HPT-JT syndrome is caused by mutations of the cell division cycle protein 73 homolog (*CDC73*) gene, at chromosome 1q31.2 [5]. The 17 exons of the *CDC73* tumor suppressor gene encode the predominantly nuclear, 531-amino acid protein, parafibromin [6]. Parafibromin regulates gene transcription as part of the RNA polymerase II-associated polymerase-associated factor 1 (PAF1) complex that has a fundamental role in chromatin remodelling [6, 7]. Parafibromin inhibits cell proliferation by blocking the expression of cyclin D1 [8] and as a component of the Wnt signalling pathway [9]. Moreover, the *CDC73* gene is implicated in sporadic parathyroid carcinomas and mutations are present in up to 70% of cases [10, 11]. Parafibromin can serve as a parathyroid carcinoma marker. While it is expressed in normal parathyroid glands, parathyroid adenoma and hyperplasia, it is usually absent in parathyroid carcinomas and some atypical adenomas [12].

The majority of the *CDC73* gene loss-of-function mutations associated with hyperparathyroidism and parathyroid carcinoma are frame-shift, nonsense or missense occurring within the protein-encoding exons [13]. Recently, some HPT-JT and FIHP cases without these types of mutation were shown to have intragenic or whole deletion of *CDC73* [14–19].

We report here a 3-generation HPT-JT syndrome family in which although initial analysis identified a variant in the 5’UTR of the mRNA no mutation was found in the protein-coding exons or exon/intron junctions. Further analysis revealed an intragenic deletion of exons 4–10 of *CDC73* that co-segregated with the 5’UTR variant in the affected individuals.

## Methods

### Patients

**Fig. 1:**
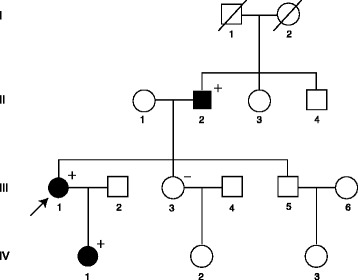
Pedigree of family with HPT-JT and *CDC73* gene mutation. Clinical status is indicated by open symbols (unaffected or status not known) and solid symbols (affected). Proband is indicated by the arrow. The presence (+) or absence (−) of the 5’UTR variant/exon 4–10 deletion in tested family members is shown

The proband (III-1, Fig. 1) was a 48-year-old woman who presented for evaluation of weight loss and fatigue. She had a history of nausea, polydipsia and polyuria but not of constipation or nephrolithiasis. Physical examination was remarkable for a cachectic appearance (BMI = 18.7 kg/m^2^) and the presence of a neck mass in the region of the right superior thyroid lobe that was hard on palpation. Laboratory tests revealed markedly elevated total serum calcium of 4.21 mmol/L (normal ≤2.60) and serum parathyroid hormone (PTH) of 133 pmol/L (normal <7.6). The patient’s hypercalcemia was emergently treated with intravenous fluids, furosemide and pamidronate.

**Fig. 2:**
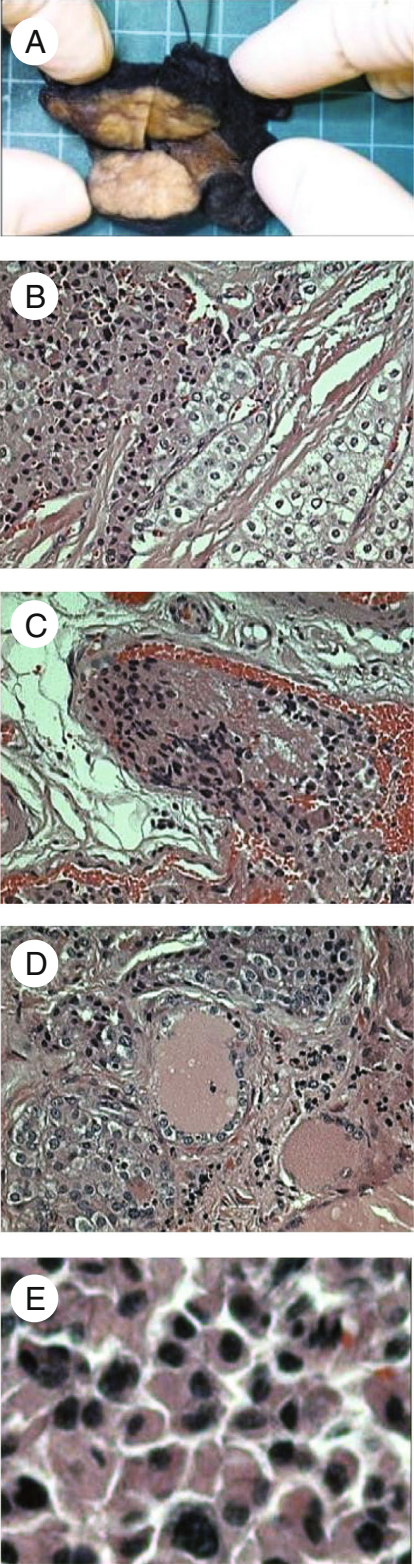
Gross view (**a**) and histology (**b**-**e**) of parathyroid carcinoma surgical specimen from proband (III-1, Fig. 1). **a** Tumor with features of fibrous banding. **b** Tumor overview. **c** Vascular invasion. **d** Thyroid invasion. **e** Nuclear atypia

Ultrasound imaging and sestamibi scan suggested the presence of ~3 cm neck mass in close proximity to the posterior aspect of the right hemithyroid lobe. Parathyroid exploration revealed a local gross invasion of the mass into surrounding structures, and a right hemithyroidectomy in addition to parathyroidectomy was performed. Pathological evaluation of the 3.5 cm surgical specimen (Fig. 2a and b) revealed vascular invasion (Fig. 2c), thyroid invasion (Fig. 2d) and nuclear atypia (Fig. 2e). Immunochemistry demonstrated PTH positivity and absence of thyroglobulin staining. A diagnosis of parathyroid carcinoma was made, and while there were no metastatic lymph nodes, the margins were positive. After surgery, the patient developed reactive hypocalcemia due to hungry bone syndrome, which was managed with oral calcium supplementation. After initial discharge she was readmitted on two occasions for marked hypocalcemia requiring intravenous or oral calcium and calcitriol. This issue had resolved by the time she received radiation therapy 3 months after surgery, but she developed radiation-induced hypothyroidism requiring oral levothyroxine replacement. Her weight improved and she is asymptomatic with no evidence of disease recurrence 6 years post-treatment.

A search for other manifestations of the HPT-JT syndrome was conducted. Dental panoramic radiograph, bone scan, renal and hepatic ultrasounds were normal. However, approximately 18 months after initial diagnosis a uterine leiomyoma [20] of 6.3 by 4.8 by 4.5 cm was found on ultrasound during work-up for menorrhagia and was treated surgically.

The patient’s first-degree family members were also evaluated. The father (II-2, Fig. 1) had a 2.2 cm parathyroid adenoma removed at the age of 32, but other manifestations of HPT-JT were not noted. The daughter (IV-1, Fig. 1) underwent screening biochemistry and was found to have PHPT (total serum calcium, 3.1 mmol/L, PTH, 8.7 pmol/L). Ultrasound imaging demonstrated a hypoechoic nodule posterior to the left hemithyroid lobe, and she underwent surgical excision of a parathyroid adenoma.

### Genetic testing

The Institutional Research Ethics Board of the IRCCS Casa Sollievo della Sofferenza Hospital approved the protocol and informed consent was obtained from the proband and family members. Genomic DNA was extracted from peripheral white blood cells using standard methods. The entire coding sequence of the *CDC73* gene including the exon-intron boundaries was sequenced by PCR amplification and direct sequencing of all the 17 exons (16 amplicons) as previously described [12]. Moreover DNA extracted from formalin fixed paraffin embedded (FFPE) tumour tissue excised from the affected daughter was screened and loss of heterozygosity (LOH) investigated as described previously [12]. No other germline DNA or FFPE tissue was available at the time of the study.

### pGL3 constructs and luciferase assay

The 184 bp 5’UTR sequence of the mRNA (encoded by the *CDC73* gene) was PCR amplified from normal human DNA with the forward primer having a HindIII and the reverse primer having a NcoI restriction site. The PCR product, after digestion with these enzymes (New England Biolabs), was cloned into the HindIII/NcoI digested pGL3 Basic vector (Promega). The NcoI site was removed and the Kozak sequence restored by specific mutagenesis to obtain the WT-5’UTR-pGL3 construct. By site-directed mutagenesis with this construct as template an additional G was introduced into the polyG (*n* = 8) tract terminating 3 bases upstream of the ATG start site to obtain the MUT-5’UTR-pGL3 construct. Sequences of mutagenesis primers and methods are provided in the Additional file 1. Correctness of the constructs was confirmed by sequencing.

Human embryonic kidney (HEK293) cells were seeded in 6 well plates in DMEM-F12 supplemented with 10% fetal bovine serum and 1% penicillin/streptomycin. Twenty-four hours later cells were transfected (Lipofectamine 2000 – Invitrogen) with increasing concentrations of pGL3 Basic, WT-5’UTR-pGL3 or MUT-5’UTR-pGL3. After 48 h, medium was removed, cells were washed, and lysed, before being scraped from the wells and collected. After vortexing for 15 s and centrifugation at 12000 rpm (2 min at 4 °C), aliquots of the supernatant were added to Luciferase Assay reagent (Promega) and the luciferase activity read in a luminometer.

### 5' UTR-parafibromin expression constructs and western blot

The 184 bp 5’UTR sequence of the mRNA (encoded by the *CDC73* gene) was PCR amplified from normal human DNA with the forward primer having an EcoRI and the reverse primer having an SgfI restriction site. The PCR product, after digestion with these enzymes, was cloned (upstream of the open reading frame) into the EcoRI/SgfI digested pCMV6 vector that expresses the human *CDC73* cDNA encoding parafibromin Myc/Flag-tagged at its COOH-terminus (Origene RC209479) [21].

The SgfI site was deleted and the 5′ UTR sequence restored by mutagenesis to generate the WT-5’UTR-Flag construct. By site-directed mutagenesis with this construct as template an additional G was introduced into the polyG (*n* = 8) tract terminating 3 bases upstream of the ATG start site to obtain the MUT-5’UTR-Flag construct. Sequences of mutagenesis primers and methods are provided in the Additional file 1. Correctness of the constructs was confirmed by sequencing.

HEK293 cells were cultured and transfected as described above with the WT-5’UTR-Flag and MUT-5’UTR-Flag constructs. Total cellular proteins were extracted in radioimmunoprecipitation (RIPA) buffer (150 mM NaCl, 50 mM Tris-HCl, 1% Nonidet P-40, 0.1% sodium dodecyl sulfate, 0.5% sodium deoxycholate, pH 8.0) supplemented with Complete EDTA-Free Protease Cocktail Inhibitor (1 tablet/10 mL RIPA). Protein aliquots were electrophoresed through 8% SDS polyacrylamide gels, electrotransferred to PVDF membrane (Millipore, Billerica, MA), blotted overnight at 4 °C with rabbit anti-Flag monoclonal antibody (Cell Signaling Technology) and for 1 h at room temperature with horseradish peroxidase-conjugated goat anti-rabbit IgG antibody (Biorad). Membranes were stripped and blotted with β-tubulin rabbit monoclonal antibody (Cell Signaling Technology). Densitometric analysis was made with ImageJ (http://rsbweb.nih.gov/ij/).

### SNP array analysis

Whole-genome copy number variation (CNV) analysis was carried out with the CytoScan HD array platform (Affymetrix, Santa Clara, CA) on leukocyte DNA of the proband (III-1, Fig. 1), her father II-1, her daughter IV-1 and her sister III-3. The array contains more than 2.6 million markers for copy number analysis and approximately 750,000 SNPs that fully genotype with greater than 99% accuracy. The CytoScanHD assay was performed according to the manufacturer’s protocol, starting with 250 ng DNA. DNA was digested with the NspI restriction enzyme, ligated to an appropriate adapter for the enzyme, and subjected to PCR amplification using a single primer. After digestion with DNase I, the PCR products were labeled with a biotinylated nucleotide analogue, using terminal deoxynucleotidyl transferase. Hybridization to the microarray was carried out in a Hybridization Oven 645 while subsequent washing and staining were performed using the Fluidics Station 450. The array was then scanned with the Scanner 3000 7G and both the quality control step and copy number analysis were performed using Chromosome Analysis Suite Software version 2.0. The raw data file (.CEL) was normalized using the default options and an unpaired analysis was performed using as baseline 270 HapMap samples to obtain the copy number value from. CEL files while the amplified and/or deleted regions were detected using a standard Hidden Markov Model (HMM) method. Base pair positions were obtained from the University of California Santa Cruz (UCSC) Genome Browser (http://genome-euro.ucsc.edu/cgi-bin/hgGateway?redirect=manual&source=genome.ucsc.edu), build GRCh37 (hg19).

### Statistics

Data are expressed as mean ± SE of triplicate estimations with each experiment repeated three times and a *p* value <0.05 was considered statistically significant.

## Results

### *CDC73* mutation screening

**Fig. 3:**
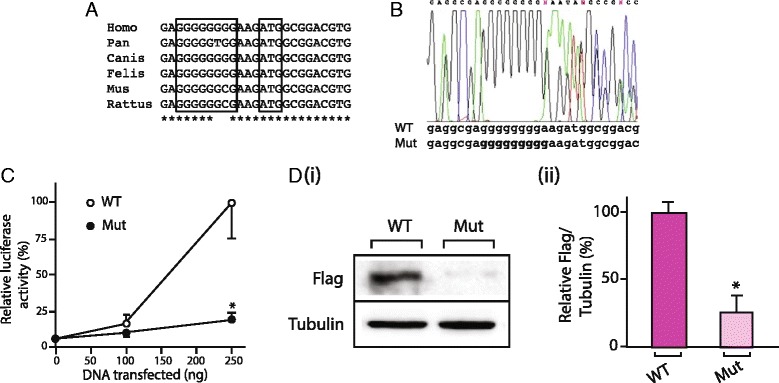
**a**. Species sequence alignment of *CDC73* encoding the proximal 5’UTR. The g tract (*n* = 8) and the ATG start codon are boxed. **b** Sequence chromatogram of leukocyte genomic DNA of proband (III-1, Fig. 1) showing heterozygosity for insertion of an additional guanidine in the tract of eight guanidines (c.–4_11insG). **c** Luciferase activity (mean ± SE) of cells transfected with either WT-5’UTR-pGL3 or MUT-5’UTR-pGL3 constructs. See text for details. *, *p* < 0.05. **d** (i) Parafibromin (Flag) and β-tubulin (Tubulin) western blots of cells transfected with either WT-5’UTR-Flag or MUT-5’UTR-Flag constructs. (ii) Densitometric analysis. **, *p* < 0.01. See text for details

**Fig. 4:**
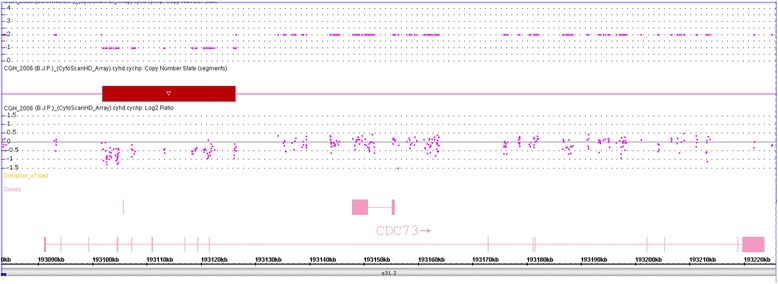
CytoScan HD Array analysis results of the patient. Intensity data (log 2 ratio value) of each probe is drawn along chromosome 1 from 193.00 to 193.22 Mb (USCS Genome Browser build February 2009, hg19). The red bar represents the 1q31.2 deletion identified, encompassing exons 4 to 10 of the CDC73 gene

*CDC73 s*equence analysis of genomic DNA of whole blood of the proband (III-I, Fig. 1) did not reveal a mutation in the coding region or at splice sites. However, within the 5’UTR an insertion of an additional guanidine within a well-conserved polyG tract (n-8), 3 bp upstream of the ATG start site, namely c.-4_-11insG, was found (Figs. 3a and b). In addition, the SNP array analysis revealed an interstitial microdeletion of 0.25 Mb in band 1q31.2 (Fig. 4) in cis with the 5’UTR variant. The hemizygous region encompassed part of the *CDC73* coding sequence from exon 4 to 10 (Fig. 4). These same changes (5′ UTR variant, gene deletion) were identified in genomic DNA of the affected father (II-2, Fig. 1) and the affected daughter (IV-1, Fig. 1) of the proband. These changes were not found in genomic DNA of the unaffected sister (III-3, Fig. 1) of the proband. Sequencing and LOH analysis of FFPE parathyroid tissue of the proband (III-1, Fig. 1) was uninformative with respect to identifying a somatic second hit involving the wild-type *CDC73* allele.

### In vitro functional analysis of the 5’UTR variant

In the first approach, HEK293 cells were transfected with the pGL3 Basic construct in which the *CDC73* 5’UTR (either wildtype or mutant) had been inserted upstream of the luciferase coding sequence. While at low concentration (50 ng) of DNA transfected luciferase activity was minimal (no different from mock-transfected cells, data not shown), with higher concentrations of DNA (250 ng) significant luciferase activity was noted with the WT-5’UTR-pGL3 construct whereas the activity of the MUT-5’UTR-pGL3 construct was significantly less (Fig. 3c).

In the second approach, HEK293 cells were transfected with an expression vector in which the wild type or mutant *CDC73* 5’UTR had been inserted upstream of a parafibromin cDNA having a Flag epitope encoded at its COOH-terminus. Western blot analysis of cell protein extracts revealed markedly reduced levels of the exogenous parafibromin in cells transfected with the MUT-5’UTR-Flag construct as compared with those transfected with the WT-5’UTR-Flag construct [Fig. 3d (i) and (ii)].

## Discussion

Here we describe a rare case of HPT-JT syndrome, in which the hyperparathyroidism in affected family members co-segregated with an altered *CDC73* allele harboring a large intragenic deletion and a 5’UTR variant. While deletion of exons *CDC73* 4–10 is clearly pathogenic, our in vitro analysis also suggests impaired function of the *CDC73* mRNA having the c.-4_-11insG alteration However, species sequence comparison (UCSC, https://genome.ucsc.edu/) reveals that the 8G–tract shows limited phylogenetic conservation from humans to rodents, and in lower species the length and sequence vary (Fig 3a). With respect to the 5’UTR variant, recently, it appeared in the ClinVar database (https://www.ncbi.nlm.nih.gov/projects/SNP/, http://www.internationalgenome.org/ and https://www.ncbi.nlm.nih.gov/clinvar/, code rs886043365 and 286,328, respectively), but it was not found in ExAC or gnomAD, (http://exac.broadinstitute.org/, http://gnomad.broadinstitute.org/) databases. In the ClinVar database, Minor Allele Frequency (MAF) was not available. Mutations were identified only 5 times in subjects affected by HPT-JT (2 cases), parathyroid carcinoma (1 case), and in 2 cases with an unreported clinical condition. The reported clinical significance was not unanimous, being “uncertain” in 3 cases and “benign or likely benign” in 2 cases.

In our family case, in which the 5’UTR variant is in *cis* with the large deletion, we believe that affected status was due to the loss of genomic sequence, without any ascribable influence of the 5’UTR variant. Further ad hoc studies and genetic testing on larger cohorts would help to clarify this issue.

Mutations of the *CDC73* gene have presented as missense/nonsense, frameshift insertion or deletion that are scattered throughout the entire coding sequence with selectivity for some exons as opposed to others [12, 13]. As such mutations have not been found in all HPT-JT syndrome cases and its variants, the search for large genomic deletions at the *CD73C* locus has recently intensified, leading to their identification in several cases [14–19] (see Table 1). In one large study [16], large gene deletions represented 35% of all the CDC73 genetic lesions identified, regardless of phenotypic presentation (sporadic parathyroid carcinoma, FIHP or HPT-JT), suggesting a possible underestimation of the presence of such genomic rearrangements at the *CDC73* locus in the pathogenicity of the syndrome.

**Table 1:** Reports of gross deletion of *CDC73*

Case #	Sex	Age (ys)	Family history	Total calcium (mmol/L)	Jaw lesion	Kidney lesion	Uterine lesion	HPT pathology	Molecular abnormality	Predicted effetc	Reference
#1	M	25	No	4.4	No	No		A	Whole gene deletion	CDC73 gene loss	Domingues et al., 2012
#2	F	18	Yes	3.6	No	No	No	A	Whole gene deletion	*CDC73* gene loss	Cascón et al., 2011
#3	M	35	Yes	3.3	Un	Yes		A	c.237 +?_308–?del ex 3	Exon 3 deletion	Bricaire et al., 2013
#4	F	16	Yes	3.1	Yes	No	No	A	c.307 +?_513–?del ex 4–6	Exon 4, 5, 6 deletion	Bricaire et al., 2013
#5	M	16	No	4.2	No	No		A	c.512 +?_1155–?del ex 7–13	Exon 7 to 13 deletion	Bricaire et al., 2013
#6	M	27	Yes	3.2	No	No		A	c.131 +?_308-?del ex 2–3	Exon 2 and 3 deletion	Bricaire et al., 2013
#7	F	37	No	4.2	Yes	Yes	No	C	1q31.1–1q31.3 del	*CDC73* gene loss	Bricaire et al., 2013
#8	F	43	No	3.1	No	No	Yes	C	Whole gene deletion	*CDC73* gene loss	Bricaire et al., 2013
#9	F	20	No	Un	Yes	Yes	Yes	C	Whole gene deletion	*CDC73* gene loss	Bricaire et al., 2013
#10	M	32	Yes	Un	No	No		C	c.1-?_972-?del ex 1–10	Exon 1 to 10 deletion	Korpi-Hyövälti et al., 2014
#11	F	15	Yes	3.1	No	No	Yes	A	c.307 +?_513–?del ex 4–6	Exon 4, 5, 6 deletion	Kong et al., 2014
#12	F	8	No	4,1	No	No	No	C	c.1-?_131-?del ex 1	Exon 1 deletion	Davidson et al., 2015
#13	F	48	Yes	4.2	No	No	Yes	C	c.307 +?_972–? del ex 4–10	Exon 4 to 10 deletion	This study

Identification of a somatic mutation in the wild-type *CDC73* allele in the tumor has been important in confirming that the “second hit” hypothesis for tumor suppressor genes applies to *CDC73* (e.g., [22]). In the present case, sequencing and LOH analysis were unsuccessful in identifying a somatic second hit. In such cases in which absent parafibromin immunostaining in the tumor has been documented, a variety of mechanisms have been proposed whereby the parafibromin expression could be reduced. These include gene inactivation by hypermethylation of the *CDC73* promoter, an event that appears to be rare or absent in HPT-JT tumors [23, 24]. Evidence of inhibition of the *CDC73* gene by a transcription factor [25] or by a microRNA suppressing the *CDC73* mRNA [26] has been provided in squamous cell carcinoma but has yet to be confirmed for parathyroid neoplasia.

The present study has important implications for the clinical management of parathyroid carcinoma as occurring sporadically or within families [27, 28]. Patients presenting with apparently sporadic parathyroid carcinomas may carry germline mutations in the *CDC73* gene, and may thus have the potential to express the HPT-JT syndrome or a variant. Family members may also be mutation carriers. *CDC73* genetic and clinical testing should be considered for the following: patients with a suspicion or diagnosis of parathyroid carcinoma; family members of patients with a diagnosis of parathyroid carcinoma; and patients with a positive family history of parathyroid tumor of any sort. For asymptomatic individuals (e.g., family members) the following is recommended: periodic biochemical screen (serum total calcium and PTH): tumor surveillance, e.g., panoramic jaw x-ray, kidney and uterine ultrasound. For family members of patients with parathyroid carcinoma periodic biochemical screen regardless of *CDC73* status is recommended.

## Conclusions

The presence of a *CDC73* mutation is associated with increased risk of parathyroid carcinoma. Nevertheless, as emphasized here and in other reports, the absence of pathogenic coding variants does not exclude large genomic deletion, the search for which should be encouraged. If known preoperatively, this information will be helpful to the surgeon in planning the extent of surgery required. As the risk of recurrence has to be weighed against the possibility of incomplete disease penetrance, the optimal extent of parathyroid surgery for benign disease in the setting of a *CDC73* mutation remains challenging and controversial [29–33].

## Additional file

Additional file 1: The file contains the sequences of the mutagenesis primers and the mutagenesis protocol. (DOCX 12 kb)

## Abbreviations

BMI: Body Mass Index
CNV: Copy Number Variations
FFPE: Formalin Fixed Paraffin Embedded
FHPT: Familial Hyperparathyroidism
HEK293: Human Embryonic Kidney 293
HMM: Hidden Markov Model
HPT-JT: Hyperparathyroidism with Jaw Tumour
MEN1: Multiple Endocrine Neoplasia 1
PAF1: Polymerase Associated Factor 1
PHPT: Primary Hyperparathyroidism
UTR: Untranslated Region
