# Erratum to: Large intragenic deletion of *CDC73* (exons 4–10) in a three-generation hyperparathyroidism-jaw tumor (HPT-JT) syndrome family

**DOI:** 10.1186/s12881-017-0459-7

**Published:** 2017-09-13

**Authors:** Vito Guarnieri, Raewyn M. Seaberg, Catherine Kelly, M. Jean Davidson, Simon Raphael, Andrew Y. Shuen, Filomena Baorda, Orazio Palumbo, Alfredo Scillitani, Geoffrey N. Hendy, David E.C. Cole

**Affiliations:** 1 Medical Genetics, IRCCS Casa Sollievo della Sofferenza Hospital, San Giovanni Rotondo, Italy; 2 Department of Otolaryngology - Head and Neck Surgery, University of Toronto, Toronto, ON Canada; 3 Department of Medicine, University of Toronto, Toronto, ON Canada; 4 Division of Endocrinology, Women’s College Hospital, Toronto, ON Canada; 5 Department of Otolaryngology, Head & Neck Surgery, Sunnybrook Health Sciences Centre, Toronto, ON Canada; 6 Department of Anatomic Pathology, Sunnybrook Health Sciences Centre, Toronto, ON Canada; 7 Departments of Laboratory Medicine and Pathobiology, Medicine and Genetics, University of Toronto, Toronto, ON Canada; 8 Endocrinology, IRCCS Casa Sollievo della Sofferenza Hospital, San Giovanni Rotondo, Italy; 9 Metabolic Disorders and Complications, McGill University Health Centre-Research Institute, Montreal, QC Canada; 10 Departments of Medicine, Physiology and Human Genetics, McGill University, Montreal, QC Canada

## Erratum

Following publication of the original article [1], the authors identified the following errors in the scientific content:

p.4, para. 3: “(1 tablet/10 mL RIPA)” should read “(1 tablet/10 mL RIPA buffer)”.

p.4, para. 4: “II-1” should read “II-2”.

Minor mistakes were also identified in the table on page 6, and in the Abbreviations section on page 7.

The original article has been corrected.
