# Supplementary material for: Large intragenic deletion of CDC73 (exons 4–10) in a three-generation hyperparathyroidism-jaw tumor (HPT-JT) syndrome family
Source: BMC Med Genet. 2017 Aug 3;18:83. doi: 10.1186/s12881-017-0445-0 (PMC5543551; doi:10.1186/s12881-017-0445-0)
Supplement: Additional file 1: — The file contains the sequences of the mutagenesis primers and the mutagenesis protocol. (DOCX 12 kb) [file 12881_2017_445_MOESM1_ESM.docx]

**Additional file 1**

Primers sequences used either for pGL3 vector and for CDC73 vector

| *pGL3 vector* | 5'-3' |
| --- | --- |
| 5UTR-pGL3-HindIII_for | ATCTAAGTAAGCTTCGGCGGC |
| 5UTR-pGL3-NcoI_rev | GCGTCTTCCATGGCTTCCCCC |
| 5UTR-pGL3WT_for | AGGCGAGGGGGGGGAAGATGGAAGACGCCAAAA |
| 5UTR-pGL3WT_rev | TTTTGGCGTCTTCCATCTTCCCCCCCCTCGCCT |
| 5UTR-pGL3-InsG_for | AGGCGAGGGGGGGGGAAGATGGAAGACGCCAAAA |
| 5UTR-pGL3-InsG_rev | TTTTGGCGTCTTCCATCTTCCCCCCCCCTCGCCT |
|  |  |
| *CDC73-Myc-Flag vector* | 5'-3' |
| 5UTR-CDC73-EcoRI_for | GTCAGTGAATTCCGGCGGCCTGGGTGGCTACTGC |
| 5UTR-CDC73-SgfI_rev | ACTGATGCGATCGCCTTCCCCCCCCTCGCCTCCGC |
| 5UTR-CDC73WT_for | AGGCGAGGGGGGGGAAGATGGCGGACGTGCTTAG |
| 5UTR-CDC73WT_rev | CTAAGCACGTCCGCCATCTTCCCCCCCCTCGCCT |
| 5UTR-CDC73-InsG_for | AGGCGAGGGGGGGGGAAGATGGCGGACGTGCTTAG |
| 5UTR-CDC73-InsG_rev | CTAAGCACGTCCGCCATCTTCCCCCCCCCTCGCCT |

**pGL3 and CDC73 expression vectors**

Mutagenesis reactions were conducted in a total volume of 50 uL containing 100 ng DNA, 5 ul 10X buffer, 125 pmol (final) of each primer, 8 uMol (final) dNTPs and 1U Pfu Taq polymerase (Promega). PCR conditions were: 95°C x 2 min, and 18 cycles of 95°C x 30 sec, 55°C x 1 min and 72 °C X 12 min. One microlitre of Dpn1 (New England Biolabs) was added to digest parental DNA and 3 uL used to transform DH5a cells (Invitrogen). Colony PCR and sequencing identified the mutated clones. Midipreps were performed with Plasmid Midi Kit (QIAGEN).
